# Role of thioredoxin in chronic obstructive pulmonary disease (COPD): a promising future target

**DOI:** 10.1186/s12931-023-02574-4

**Published:** 2023-11-24

**Authors:** Heena Kansal, Vishal Chopra, Kranti Garg, Siddharth Sharma

**Affiliations:** 1 Department of Biotechnology, Thapar Institute of Engineering and Technology, Patiala, India; 2 Department of Pulmonary Medicine, Government Medical College, Patiala, India

**Keywords:** COPD, Thioredoxin, Oxidative stress, Inflammation, ROS

## Abstract

**Introduction:**

Thioredoxin (Trx) is a secretory protein that acts as an antioxidant, redox regulator, anti-allergic, and anti-inflammatory molecule. It has been used to treat dermatitis and inflammation of the digestive tract. In the lungs, Trx has a significant anti-inflammatory impact. On the other hand, Chronic Obstructive Pulmonary Disease (COPD) is one of the significant causes of death in the developed world, with a tremendous individual and socioeconomic impact. Despite new initiatives and endless treatment trials, COPD incidence and death will likely escalate in the coming decades.

**Areas covered:**

COPD is a chronic inflammatory disease impacting the airways, lung parenchyma, and pulmonary vasculature. Oxidative stress and protease-antiprotease imbalances are thought to be involved in the process. The most popular respiratory inflammatory and allergic disorders therapies are corticosteroids and β-receptor agonists. These medications are helpful but have some drawbacks, such as infection and immunosuppression; thus, addressing Trx signalling treatments may be a viable COPD treatment approach. This review shall cover the pathophysiology of COPD, the pharmacognosy of anti-COPD drugs, including the assets and liabilities of each, and the role and mechanism of Trx in COPD treatment.

**Expert opinion:**

Limited research has targeted the thioredoxin system as an anti-COPD drug. Spectating the increase in the mortality rates of COPD, this review article would be an interesting one to research.

## Introduction

COPD is an avoidable illness marked by concurrent partially reversible airflow restriction and an inappropriate inflammatory reaction of the lungs to harmful particles or gases [1]. Many studies have shown that a subset of smokers has a higher rate of decline in maximal forced expiratory flow, as indicated by the forced expiratory volume (FEV1) and FEV1/FVC (Forced vital capacity) ratio, which are necessary for normal lung functioning [2]. Emphysema, a condition characterised by the loss of lung elasticity and small airway obstruction, a common feature of COPD, contributes to decreased FEV1 and FVC [3].

The radial traction of the alveolar walls and their external surface is crucial in keeping the airways within the lungs open. However, in emphysema, where there is a lack of elastic recoil, the increase in lung volume leads to enclosure. This enclosure is exacerbated by small airway obstructions, as the surface tension at the air–liquid interface within the alveoli is heightened due to the restricted airway space. Additionally, the reduced shortening of the airway smooth muscles required for airway closure further contributes to this issue [4]. Spirometry is a test that helps in the timely screening of COPD patients suffering from persistent obstruction. However, no or very mild symptoms may be difficult to detect through spirometry before critical deterioration. Hence, understanding the various COPD individuals' lung-function dynamics is vital to avoid the overtreatment of preclinical-COPD or pre-COPD individuals [5]. The current standard care is to treat the consequences of airflow restriction-induced mostly by the symptoms indicated earlier to alleviate the ensuing dyspnea. But presently, no method exists to stop the malady from progressing [6]. Bronchodilators and β-receptor agonists are common treatments, although they have many side effects [7]. The pathophysiology of COPD, the pharmacognosy of anti-COPD medications, and their drawbacks shall be discussed in this article. It focuses on the role and mechanism of Thioredoxin (Trx) in treating COPD.

## Altered pathways and mechanisms involved in COPD pathogenesis

### Oxidant-antioxidant imbalance in COPD

**Fig. 1:**
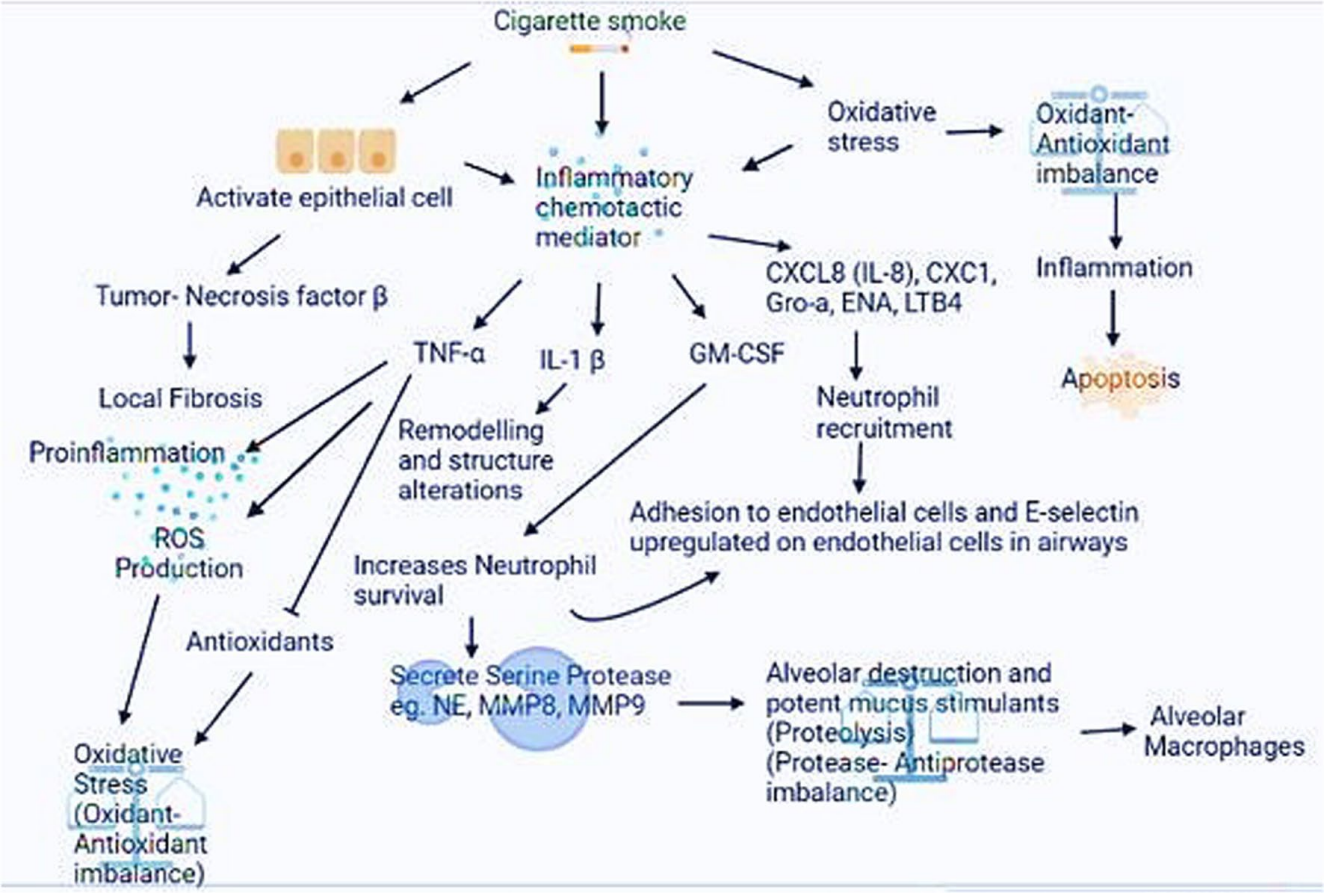
Pathophysiology of COPD. Chronic smoking (CS), which causes oxidative (OS) stress, proteolysis, and chronic inflammation, is linked to COPD development. CS causes a substantial amount of reactive (ROS) oxygen species, disrupting the harmony between free radicals and antioxidants, intensifying inflammatory responses, and accelerating lung cell damage

Reactive oxygen species (ROS) such as O_2_^−^, OH^−^, and H_2_O_2_ are formed when oxygen reacts with excess electrons in the body [8]. Many key physiological activities like gene transcription, immunological response, and signalling transduction are influenced by ROS. ROS and RNS produced by cellular and environmental sources may enter the lungs and end up with many respiratory diseases like COPD, Asthma, Pneumonia, Respiratory (RDS) distress syndrome, and COVID-19 [9]. Excess ROS, also known as oxidative stress (OS), is considered one of the major causes of COPD. Multiple structural and inflammatory cells of the airways can also produce RNS/ROS as cigarettes contain approximately 1016–1017 oxidants every puff and about 4700 compounds, including nitrogen oxides, superoxide radicals, and peroxynitrite; they can cause OS in the lower airways.The gas phase contains 1015 inorganic and organic radicals per puff, including nitric oxide (NO·), nitrogen dioxide, and ONOO. One of the main RNS (responsible fpr nitrosative stress) and ROS is NO·. RNS/ROS can also be produced via multiple structural and inflammatory cells of the airways. Endogenously, the production of oxidants occurs mainly in mitochondria, one of the primary endogenous sources of ROS. Up to 12 sites, divided into two subgroups: the reduced nicotinamide adenine dinucleotide/nicotinamide adenine dinucleotide (NADH/NAD +) isopotential group and the ubiquinol/reduced ubiquinone (UQH2/UQ) isopotential group, are potential sources of mitochondrial ROS. NADPH (NOX) oxidase, cytochrome P-450, XO, and peroxisomal enzymes are some endogenous pro-oxidant enzymes that produce ROS in mammals [10]. Our body has defence mechanisms divided into two categories: enzymatic or nonenzymatic, which serve as the body's principal barrier against reactive oxygen and reactive nitrogen species. These mechanisms help to counteract the effects of oxidative stress. Low molecular weight substances like GSH, ascorbate, urate, alpha-tocopherol, bilirubin, and lipoic acid are nonenzymatic antioxidants. In the lungs, these nonenzymatic antioxidant concentrations differ. Antioxidants, whether made in the body or provided outside, can scavenge ROS and lower the oxidation of cellular components, resulting in reduced oxidative stress [11]. Cigarette (CS) smoke is a significant risk factor in COPD, but other genetic and environmental factors also play an essential role. The airway epithelium, which acts as a barrier to inhaled hazardous substances, is the principal target of inhaled cigarette smoke [12]. COPD patients have higher ROS generation and oxidative stress levels, implying that their endogenous antioxidants may not be enough to protect them from oxidative damage caused by cigarette smoke [13]. The pathophysiology of COPD has been shown in Fig. 1. Many studies have shown that COPD patients’ antioxidant levels are significantly lower [14]. So, utilizing antioxidants or increasing endogenous antioxidants to reduce oxidative stress could be an available treatment approach. However, finding COPD antioxidants that are both effective and reliable has been difficult, owing to the high oxidative stress levels in the lungs. Treatment aims to re-establish a normal redox status within the lungs while keeping the advantages of oxidant signalling. Few biomarkers may determine which patients would respond better to antioxidant therapy and how many doses are required to restore redox equilibrium in COPD patients [15].

### Protease-antiprotease imbalance in COPD

**Fig. 2:**
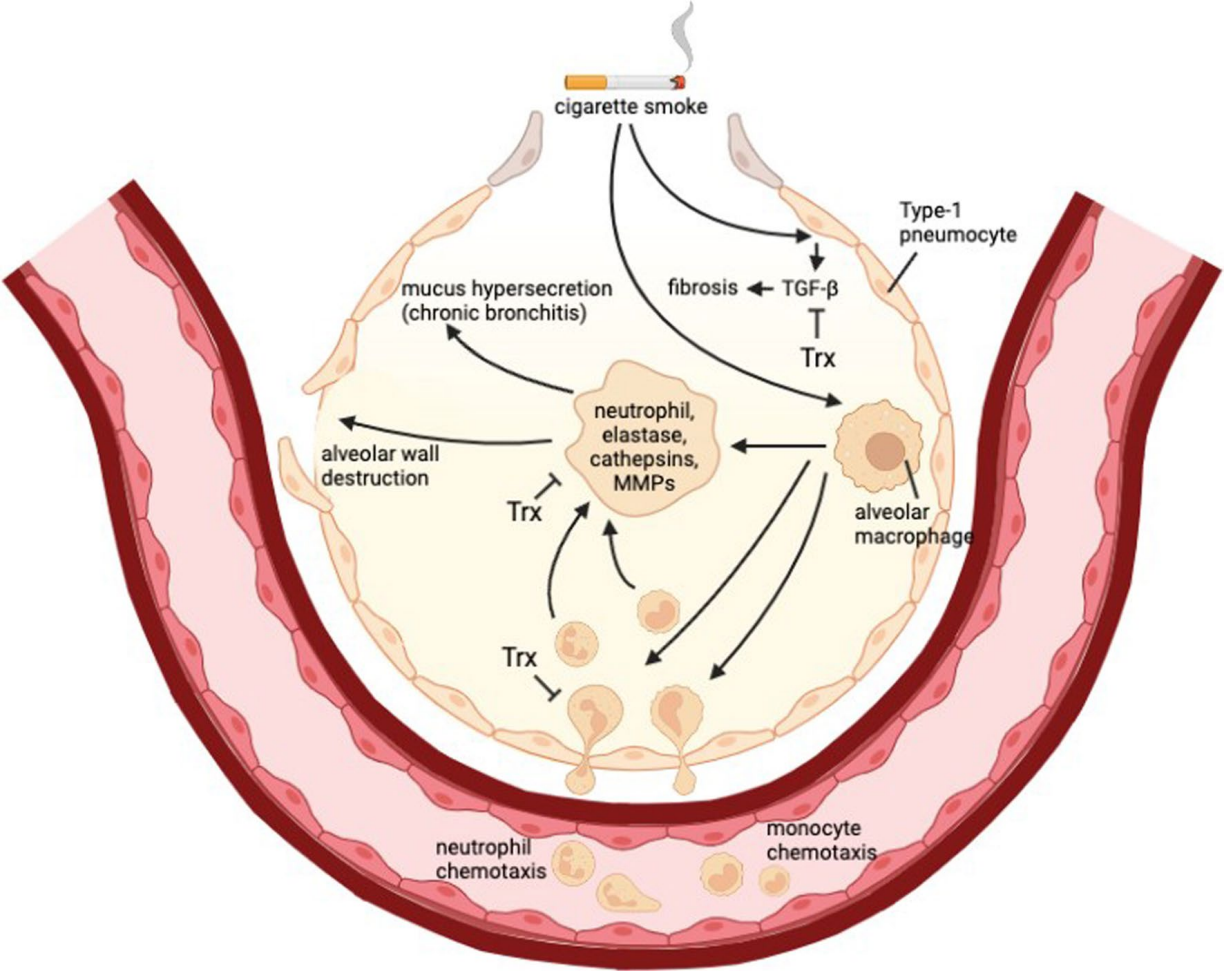
Proposed pathway of thioredoxin inhibiting recruitment of inflammatory cells and proteases. Smoking and other irritants cause epithelial cells and macrophages to start releasing a number of chemotactic substances that draw inflammatory cells to the lungs. These substances include CCL2, which attracts monocytes by acting on CCR2, CXCL1, and CXCL8, which attract neutrophils. Along with macrophages and epithelial cells, these inflammatory cells release proteases like MMP-9 that break down elastin and lead to emphysema. Mucus hypersecretion is also brought on by neutrophil elastase. TGFβ- is also released by macrophages and epithelial cells, which promotes the growth of fibroblasts and causes fibrosis in the lower airways. Many studies have revealed a significant role of thioredoxin in regulating this pathway

Proteases serve a crucial function in the protection of the respiratory host. Proteases and antiproteases work together to keep tissue homeostasis in a healthy lung, and their imbalance makes the lung parenchyma more susceptible to protease-mediated damage [16]. Proteases and their by-products are also known to have pro-inflammatory effects, and people with COPD have higher amounts of these in their lungs. Lungs are abundant in proteolytic enzymes such as matrix (MMPs) metalloproteinases, serine proteases, and cysteine proteases, out of which serine proteases are the most prevalent. The four respiratory antiprotease antagonists to these are metallopeptidase (TIMPs) inhibitors, secretory leukocyte protease inhibitor (SLPI), and serine protease (serpins) inhibitors, including trapping-2/elafin. Each class’s target substrates, cellular sources, and active sites are distinct [17]. Alpha-1-antitrypsin (α1AT) deprivation (a serine protease blocker that modulates neutrophilic cell migration, especially FcγRIIIb and CXCR1 signalling) is one of COPD's most common hereditary causes [18]. In addition, in individuals with an α1AT deficiency, when medicated with α1AT augmentation treatment, α1AT has been demonstrated to govern the overall metalloproteinase-2 (MMP2) and cathepsin B level [19]. Too much proteolysis happens with prolonged tobacco use and is responsible for bronchiectasis and emphysema, as shown in Figs. 1 and 2. As a result, maintaining the protease–antiprotease equilibrium in the lungs could prevent parenchymal damage and reduce chronic airway inflammation [20].

### Dysregulated NF-κB signalling in COPD

Adhesion molecules, chemokines, and cytokines are the pre-inflammatory genes regulated by the nuclear factor Nuclear factor kappa B (NF-κB) pathway, traditionally recognized as an archetypal pro-inflammatory signalling system [21]. The two NF-κB (p52 and p50, and its precursors p100 and p105, respectively) subfamily proteins and the three REL (p65/RELA, c-REL, and c-RELB) subfamily proteins make up the NF-κB (nuclear factor kappa-light-chain-enhancer of activated B cells) transcription factor [22]. The non-canonical and canonical (also known as the conventional pathway) signalling pathways that activate NF-κB targeted genes have been identified. In the canonical pathway, cytokine (TNFR, IL-1R) receptors and pattern recognition receptors such as toll-like receptors activate p50, a p105 product usually associated with RelA (or c–Rel). The non-canonical route, which includes p52 and RelB, is aggravated by the lymphotoxin receptor, *i.e.,* CD40 and B cell-activating factor receptor three. The most prevalent form of NF-κB is p50/p65 (RelA), a heterodimer in the cytoplasm [23].

The stimulation of NF-κB, for example, is mediated by oxidants [24]. NFkB activity in BAL macrophage epithelial cells is higher in COPD patients, and NF-κB activation increases even more during exacerbations [25]. The chaperone protein, Ikβα, limits NFkB activation by masking the NLS region of NF-κB in the cytoplasm [26]. In COPD patients, inhibition of IKK2 selectively inhibits IK-B phosphorylation, preventing NFkB stimulation, including its nuclear translocation and the inflammatory reaction, but it was observed that IKK2-deficient mice were not alive. At the same time, B-cells from conditional IKK2 deficient mice showed significantly reduced proliferation when injected with LPS, anti-CD40, or anti-IgM stimulation (messengers that stimulate NFκB under standard conditions). NAC and HDAC have been demonstrated to block NF-κB activity in intact cells, regulating cellular genes coding intracellular adhesion molecules [27].

### Dysregulation of PI3-AKT pathway in COPD

**Fig. 3:**
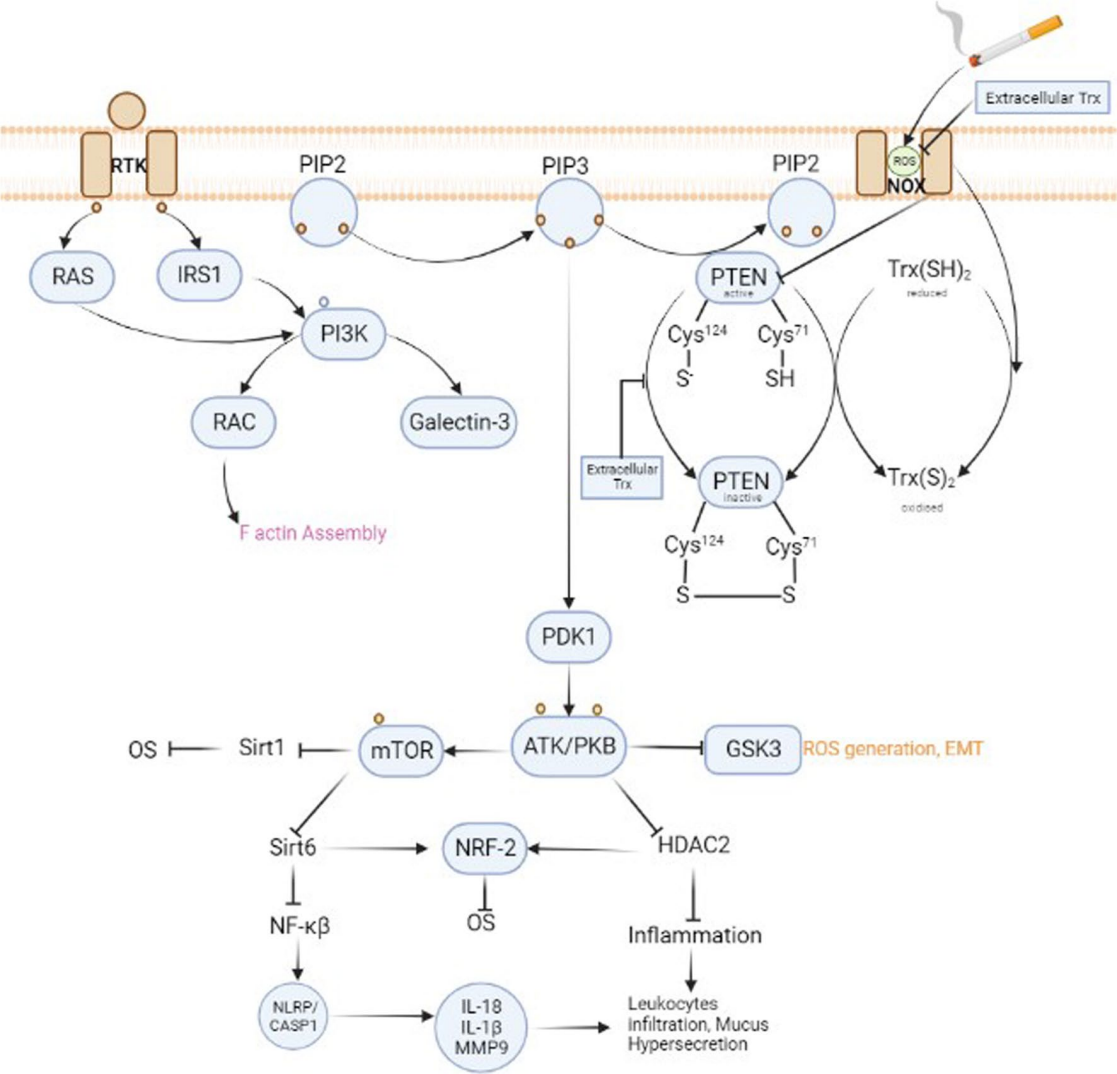
Thioredoxin reactivating PTEN. PTEN is an antagonist of PIP3. PTEN is deactivated under oxidative stress, which triggers the PDK1 pathway. Thioredoxin is also dimerized during oxidative stress or can form a dimer with PTEN. However, extracellular thioredoxin has the ability to restore the redox equilibrium of the cell, thereby activating PTEN once more and inhibiting the pi3-apk pathway

The lipid kinase (PI3K) phosphatidylinositol-3-kinase form [PI (3, 4, 5) P3] phosphatidylinositol-3,4,5-trisphosphate [PI (3, 4, 5) P3]. It is necessary for Akt transportation to the plasma membrane, which is phosphorylated and stimulated by PDK 1 and 2 (phosphoinositide-dependent kinase). PI3K signalling is crucial for mediating numerous biological responses in distinct developmental and tissue contexts, including cell growth, survival, growth, proliferation, differentiation, DNA repair, and apoptosis. As shown in Fig. 3, Akt, commonly called protein kinase B, is PIP3’s primary target. Threonine308 and Serine473 residues get phosphorylated by the [28] PDK1 and the mammalian target (mTORC2) of rapamycin complex 2 [29].

Akt also increases OS by suppressing Sirtulin1 and Sirtulin6, which regulates the ratio of oxidants and antioxidants by activating mTOR. Another histone deacetylase, HDAC2 and GSK3, is also inhibited by Akt, which increases inflammation and oxidative stress. FOXO (transcription factor) cannot activate the descending pro-apoptotic proteins by promoting itself in the cytoplasm but stimulates cell growth and proliferation when phosphorylated by Akt [30]. PI3K and its descending mediators are elevated as the lungs and airways change in COPD. During COPD progression, the wide range of expression of PI3K signalling molecules suggests that they are dynamically regulated in this disease. Any disturbance in PI3K signalling influences the standard functioning of alveolar immune and airway epithelial cells, resulting in increased immunological response. A hallmark of COPD, chronic inflammation is caused by an abnormally increased immune response. In contrast to typical growth hormones, extrinsic stresses can also cause enhanced activation of PI3K signalling. Many studies have found that PI3K and (Phosphodiesterase-4)PDE4 are elevated in COPD patients, while Phosphate and tensin homologue (PTEN)(suppressor for PIP3) is downregulated [31–33].

## Present treatments and limitations of COPD

COPD, a result of interactions between genes, environment, and lifespan, is commonly treated only for symptom relief. Unfortunately, there are currently no treatments that consistently improve lung function or mortality rates. Smoking cessation is the only intervention that slows the decline in lung function [34]. Bronchodilators, corticosteroids, and long-acting anticholinergic drugs improve lung function (FEV1), life quality, and exacerbation rates only somewhat. The relative ineffectiveness of such treatments for non-COPD may not be surprising given that (I) these pharmacological drugs are essentially “stolen” from asthma therapy, implying an underlying but likely inaccurate assumption that the diseases’ processes are comparable, and (II) A lack of responsiveness to these medicines is what defines COPD. The lack of a response to these drugs could be evidence of fundamentally different pathophysiology of the airways in COPD. The following sections outline some COPD treatment strategies [35, 36]. Some of them are shown in Table 1 [37]. Many studies proved that antioxidants (thiol derivatives, Superoxide Dismutase) SOD and (Glutathione Peroxidase) GPx memetics, mitochondria-targeted) had an inhibitory effect on oxidative s [38], and those are mentioned below.

**Table 1:** Present treatments for COPD

CLASS	ROLE	DRUG (BRAND)	ADVERSE EFFECTS
Short-acting bronchodilators	Open the airways to make breathing easier	Albuterol (Proair HFA, Ventolin HFA) levalbuterol (Xopenex) ipratropium (Atrovent HFA) Albuterol/ipratropium (Combivent Respimat)	Dry mouth, headache, cough, tremors (shaking), nervousness, a fast heartbeat
Corticosteroids	Reduces inflammation and Makes air flow easier in the lungs	Fluticasone (Flovent) Budesonide (Pulmicort) Prednisolone	Headache, sore throat, voice changes, nausea, cold-like symptoms, thrush, muscle weakness, upset stomach, and weight gain
Methylxanthines	Works as an anti-inflammatory drug and relaxes the muscles in the airways	Theophylline	Nausea or vomiting, tremors headache, trouble sleeping
Long-acting bronchodilators	Work gradually to help ease breathing	Aclidinium (Tudorza) Arformoterol (Brovana) Formoterol (Foradil, Perforomist) Glycopyrrolate (Seebri Neohaler, Lonhala Magnair) Indacaterol (Arcapta) Olodaterol (Striverdi Respimat) Revefenacin (Yupelri) Salmeterol (Serevent) Tiotropium (Spiriva) Umeclidinium (Incruse Ellipta)	Dry mouth, dizziness, tremors, runny nose, irritated or scratchy throat, upset stomach, blurry vision, rapid or irregular heart rate,an allergic reaction with rash or swelling
Combination drugs LABA/LAMA	Relief from shortness of breath	aclidinium/formoterol (Duaklir) glycopyrrolate/formoterol (Bevespi Aerosphere) tiotropium/olodaterol (Stiolto Respimat) umeclidinium/vilanterol (Anoro Ellipta)	Dry mouth, dizziness, tremors runny nose, nausea or vomiting, tremors
Phosphodiesterase-4 inhibitor	Relieve inflammation and improve air flow to lungs	Roflumilast (Daliresp)	Weight loss, diarrhea, headache, nausea, cramps, tremors, insomnia
Mucoactive drugs	Reduces mucus or thin it	Carbocysteine Erdosteine N-acetylcysteine	Nausea, vomiting, stomach pain

### Thiol derivatives

In protein structure, thiol moieties are prevalent functional groups [39]. Drugs with thiol moiety (-SH) have inhibited COPD exacerbations. They affect several biological systems, out of which (NAC) N-acetyl-L-cysteine, S-carboxymethyl-L-cysteine, and erdosteiene are primarily used [40]. The presence of a free sulfhydryl group (–SH) opens up the disulfide bonds (S–S) of the mucus high molecular weight glycoproteins, which no longer engage in crosslinking in the mucus gel layer, lowering mucus elasticity and a viscosity [41]. NAC can also increase GSH production as GSH is produced by a two-step ATP-dependent enzymatic process with Cys as the primary substrate. GSH can also be given orally or by inhalation, but Cys oxidises quickly in solution, resulting in inactive Cys–Cys [40]. Many clinical trials are performed on NAC and carbocystiene (mucolytic agents), proving that high dosages can control COPD exacerbations and that even high dosages are tolerant [42].

Thiol derivatives limitations: One issue with utilizing thiol-based antioxidants includes the high level of oxidative stress that may rapidly inactivate them in COPD lungs because of their thiol structure [42]. Despite its significance in the clinical care of COPD, NAC remains long debated due to its limited availability in an oral form and its acidic characteristics, preventing it from being inhaled. Although the lysine derivative of NAC called nacystelyn has been administered via nebulisation and a dry powder inhaler, there is no strong indication indicating antioxidant or anti-inflammatory effects in COPD. The cellular absorption rates and NAC deactylstion may not be sufficient to sustain enough Cys amounts for endogenous GSH production.However, a direct impact on oxidative stress is improbable because the rate constants for its reactivity with significant biological oxidants such as O2 and H2O2 are too low compared to the rate constants of GSH with H_2_O_2_. Also, inclined levels of circulating GSH may be beneficial. However, limited GSH absorption is due to the action of an intestinal enzyme, γ-glutamyl transpeptidase, which regenerates GSH precursors and may preclude significant intact GSH absorption from oral supplementation. GSH oral supplementation is troublesome due to its short apparent half-life and dependency on the expression of the transporter for uptake and transport into the bronchoalveolar lavage fluid (BALF). Furthermore, the massive doses required to revive GSH levels effectively result in toxicity [43–45].

### Enzyme mimetics

Catalytic enzymatic antioxidants mimic and reduce oxidative stress by reactivating reduced intracellular antioxidant enzymes like GPx and SOD. Catalytic antioxidant enzyme mimetics attenuate oxidative stress by restoring the action of depleted intracellular antioxidant enzymes, such as SOD and GPx [46]. Three types of enzyme mimics are described. M40414 and M40401 are two manganese-based macrocyclic ligands belonging to the first class of superoxide dismutase (SOD) enzyme mimics [47]. Recent research indicates that medicines that imitate Gpx-1 activities (e.g., ebselen, a selenium-based organic compound) in CS-induced inflammatory responses may have therapeutic potential in COPD patients. SOD mimetics like AEOL 10150 significantly reduced lung inflammatory reaction in rats after CS exposure when equated to a vehicle-treated control gro [47]. (MPO) Myeloperoxidase is an antibacterial iron-containing enzyme executed by neutrophil azurophilic granules and in COPD patients have considerably had much more MPO in their sputum and (BALF) bronchoalveolar lavage than controls [48].

Enzyme Mimetics Limitations: Catalase and sod mimics have rate constants of several orders of magnitude lesser than the enzymes. As a result, when they enter cells, their significance to cytosolic antioxidant defence is limited. However, catalase and SOD analogs seem to be efficient in extracellular regions where the quantities of antioxidant enzymes and the substrate are very low in number or absent. Some of the imitators may also be beneficial in the matrix of the mitochondria. However, they may behave as pro-oxidants rather than mitochondrial function protectors [49].

### MMP inhibitors

TIMPs, endogenous inhibitors of activated MMPs (calcium and zinc-dependent endopeptidases involved in extracellular (ECM) matrix remodeling), are carefully controlled [50]. TIMPs’ C-terminal and N-terminal domains buckle into two domains parts, with the N-terminal domain adequate for MMP inhibition. Except for TIMP-1, which is thought to be a weak antagonist of MT-MMPs, all TIMPs block MMP activity in non-selectively [51]. MMP-TIMP is a non-covalent tight complex with a 1:1 ratio [52]. MMPs are inhibited by TIMPs through immediate interaction with the catalytic Zn^2+^ ion through the N-terminal cysteine residue and extensive protein–protein surface contacts. MMP inhibition by its pro-domain and TIMP has a simple principle. It is a dual interconnection involving significant p-p interactions and metal ligation to the catalytic zinc ion of MMPs. TIMPs are shown to inhibit MMPs that have been investigated thus far (excluding TIMP1, which failed to block MT1-MMP). [53]. Because MMP-9 is overexpressed in COPD patients' alveolar macrophages [54]and is the predominant elastolytic enzyme produced by these cells, a specific MMP-9 inhibitor could be efficacious in preventing emphysema [55].

MMP inhibitors limitations: Non-selective MMP inhibitors like marimastat appear to produce significant musculoskeletal side effects, implying that isoenzyme-selective antagonists or inhaled administration may be required [56]. The failure of broad-spectrum MMPIs underlined the need for selective inhibitors to distinguish between various MMP family members [57] fully. More selective inhibitors are being sought, and adverse effects could be minimized by enhancing specificity for specific MMPs or focusing on transport to the lung parenchyma. The first synthetic MMP antagonists were peptidic derivatives with a hydroxamic acid Zn^2+^ binding group that chelated the Zn^2+^ ion and imitated the recognition site of natural MMP substrates like collagen. As explained below, many of these broad-spectrum MMP inhibitors considerably reduce lung damage and inflammation and thus have therapeutic potential. However, selective targeting of only the MMPs that cause the pathogenesis is a more clinically suitable method, particularly when long-term treatment is required, to avoid off-target consequences. MMP inhibitors of the next generation are no longer confined to substrate-like compounds and can have a diversity of peptidomimetic and non-peptidomimetic structures. Avoiding heavy metal chelators is preferable for attaining more specific MMP inhibition. As a result, the newest version of extremely selective MMP inhibitors lacks a zinc-binding group and instead takes advantage of MMPs' deep S19 cavity. REGA-3G12, an immunoglobulin that preferentially inhibits human MMP9, is an example of a monoclonal antibody against MMPs that appears to be a promising alternative to synthetic inhibitors. However, the difficulties of manufacturing high-molecular-weight proteins and the parenteral delivery requirements restrict their therapeutic potential [58, 59].

### NFkB inhibitors

Overexpression or prevention of Ikβα degradation, like aiming IkB ubiquitin ligase, blocking of other kinases except for IKKs, or suppression of non-canonical NFκB activity by suppressing NFkB inducing kinase are all options to inhibit IKK2 to diminish NFκB stimulation. Another treatment technique utilizes small (siRNAs) interfering RNAs or 'decoy' oligonucleotides to impede NFkB-induced gene transcription to regulate the expression of specific genes governed by NFκB. IKK2 inhibitors are considered effective anti-inflammatory medicines, including IMD-0354, BMS-345541, IMD 0650, SC-514, ACHP, PS-1145, and AS602868 Bay 65–1942. While no clinical trials employing IKK2 inhibitors have been conducted, in vitro IKK2 blockers diminish NFκB signaling largely induced by TNFα and viral exposure, including the expression of NFκB-dependent genes like ICAM-1, COX-2, Interleukin-8, IP-10, RANTES I-TAC [60]. Also, in a study, it has been seen that inhibiting 1,8-cineole, NF-κB, at noteworthy plasma concentration (1.5 g/ml) significantly hindered cytokines (tumor necrosis (TNF)factor-alpha, interleukin (IL)-1, and (IL-6, IL-8) systemic inflammation responsible for exacerbation in normal human monocyte LPS-stimulated cytokines [61].

NFkB inhibitors limitations: NFκB activity in animals has been shown to have various adverse effects, like greater susceptibility to infections with liver damage. These possible side effects will need to be examined before human research begins [62]. Histone deacetylases HDAC controls NF-κB, but HDAC has also been altered by protein nitration or carbonylation because of oxidative stress. Furthermore, NF-κB lacking animals are more susceptible to sepsis. Therefore, long-term NF-κB inhibition must be considered for immunosuppression and infection susceptibility problems [63].

### PI3-Akt inhibitors

Impairment of Akt activity also has ramifications for many critical cellular activities, making Akt a promising therapeutic target. So, it will be advantageous to develop highly targeted medicines that either restrict or restore Akt activation in distinct tissue compartments [64, 65]. The dual-specificity protein phosphatase, (PTEN), and which facilitate the dephosphorylation of PIP3 into PIP2, directly oppose the enzymatic activity of PI3K. Hence, PTEN is an antagonist for PI3K activation and is thus regarded as a crucial potential option in malignancies and several other disorders wherein PI3K signaling is a critical driver [66]. Roflumilast has been demonstrated to inhibit PDE4, whereas macrolides, GSK2269557, and Theophylline have been shown to inhibit PDE4 [67, 68].

PI3-Akt inhibitors limitations: PI3K/AKT/mTOR, as mentioned above, plays an essential role in controlling healthy growth and sustaining organ homeostasis and performance, and inhibiting this pathway could have various negative consequences. Bone marrow suppression, hyperglycemia, pneumonitis, hyperlipidemia, hepatotoxicity, and stomatitis are only a few clinical side effects linked with PI3K/AKT/mTOR inhibition. Because the roles of PI3K isoforms do not overlap, blockage of distinct isoforms generally results in different side effects. Hyperglycaemia is a frequently reported hematologic toxicity of PI3K antagonists, but it rarely occurs in patients medicated with PI3Kβ, γ, δ, or inhibitors; hyperlipidemia is peculiar toxicity of mTOR inhibitors, and rash frequently occurs in patients medicated with other PI3K inhibitors, except in those medicated with PI3Kβ inhibitors [69].

## Thioredoxin

The thioredoxin (Trx) system is one of the central antioxidant systems in mammalian cells, maintaining a reducing environment. Thioredoxin-1 (Trx1), a redox-active dithiol, is an essential regulator of cellular redox homeostasis. It is a ubiquitous redox protein present from archaea to man, performing its function by being a part of the Thioredoxin system, which contains thioredoxin itself, thioredoxin reductase, and NADPH [70]. It was earlier identified as H-donor to ribonucleotide reductase, an enzyme pivotal in DNA synthesis [71].

It is an essential antioxidant protein released by cells in response to stress to protect cells from oxidative damage caused by microbial invasion and physical or chemical stimulation. Trx system acts as dithiol-disulfide oxidoreductase, catalyzing the reduction of disulfides of proteins at a rate higher than other antioxidants like GSH or dithiothreitols, protecting cytosolic protein from aggregation or inactivation via oxidative formation of intra or intermolecular disulfides which also helps in maintaining reducing environment inside the cell [72].

Trx was first identified in bacteria forty years ago, but it has only recently been realised how many and what kind of activities it affects in human cells. Trx1 is induced by various stress factors, such as chemical exposure, infection, inflammation, metabolic malfunction, and oxidative damage. In treating various human inflammatory illnesses in animal models, it has shown exceptional anti-inflammatory and immunomodulatory properties [73].

Thioredoxin family: The Trx family is divided into two groups: Group I, which contains proteins that only encode a Trx domain, i.e., CXXC motif, exposed on the protein surface, and Group II, which contains Trx fusion proteins with some other domains. Group One Thioredoxin is a relatively tiny ancient protein responsible for changing the redox status of target proteins by reversibly oxidizing an active site dithiol located in a CXXC motif on the protein's surface. Thioredoxin breaks disulfide bonds in proteins, forming a disulfide bond at its active site. Thioredoxin reductase uses reducing equivalents obtained from NADPH or ferredoxins to convert oxidized Trx to the active form [74]. Trx modulates the activity of at least 30 target proteins, including enzymes and transcription factors, by changing their redox status. It also aids in protecting against OS by neutralizing the hydrogen peroxide, including many other radicals, directly and acting as a reducing agent for the peroxiredoxins.

**Fig. 4:**
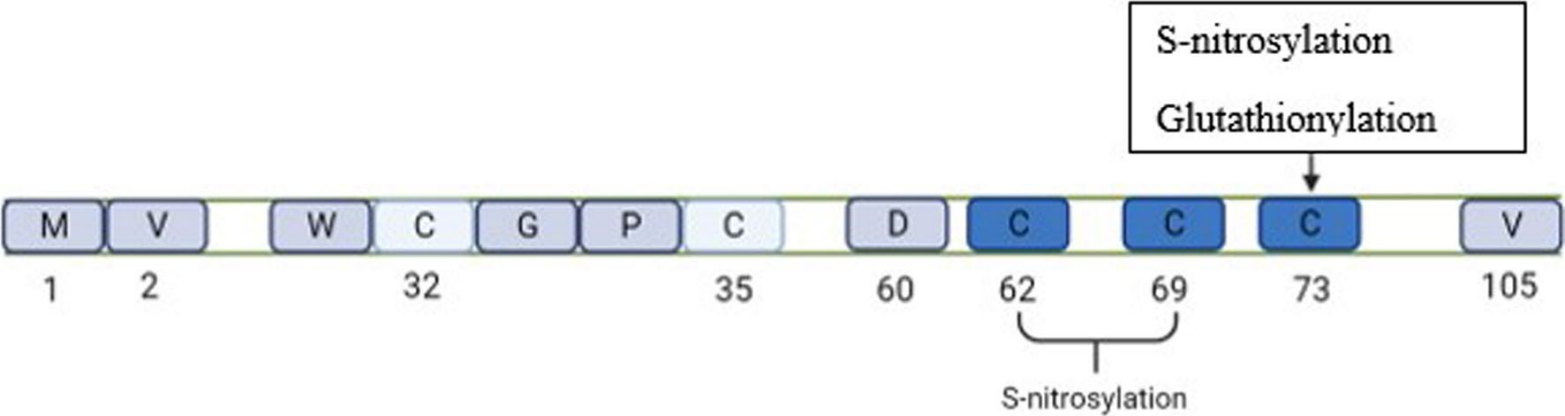
Conserved Cysteine residues in the thioredoxin active site sequence

Isoforms of thioredoxin: There are two main isoforms of Trx in mammalian cells: Trx1 and Trx2. Trx1 is the prototypical member of this family and is mainly localized in the cytosol but can be translocated into the nucleus upon stress conditions or secreted out of the cell under certain circumstances. Trx2 is the mitochondrial-restricted isoform of Trx. In addition to the Cys–Gly–Pro–Cys active site, Trx2 has an extra N-terminal mitochondrial translocation signal peptide that has been implicated in playing an essential role in mitochondria-mediated apoptosis. Besides the two conserved cysteine residues in the active site, human Trx1 has three additional cysteine residues, Cys62, Cys69, and Cys73, as shown in Fig. 4, which are absent in Trx2. These three cysteines are involved in protein transnitrosylation and denitrosylation modulation. Cys69 S-nitrosylation is involved in endothelial cells’ redox regulation and antiapoptotic functions [75]. Trx2 is also distinct from Trx1 by the fact that Trx2 participates in ROS detoxification through mitochondrial-specific peroxiredoxins and is reduced by its reductase, TrxR2, thereby constituting a functionally separate Trx system restricted to the mitochondria [76].

### Genes of thioredoxin

**Fig. 5:**
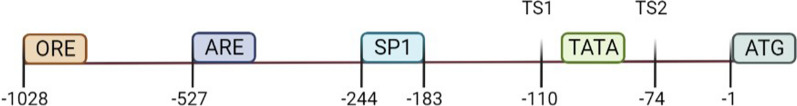
Promotor region of human thioredoxin. Three Sp1 (specificity protein 1)-binding sites are present between − 244 bp and − 183 bp, along with the first transcription start site (TS1), which is positioned at − 110 bp. With a TATA box at − 102 bp, the TS2 is only at − 74 bp. antioxidant responsive element (ARE), oxidative stress responsive element (ORE)

Trx, in humans, is coded by two different genes. Trx1 (9q31.3 having five exons and four introns) is located in a cytosolic region containing a 32cys-gly-pro-cys35 active site, conserved from E. coli to humans. Its coding region spans over 13 kb, and its promoter contains two transcriptions start sites as depicted in Fig. 5 (Trx1 encodes two transcripts of 85aa (have two isoforms) and 105aa (have one isoform) in length. Conversely, Trx2 (22q12.3 having four exons) is a mitochondrial or nuclear Trx. It encodes two transcripts, each containing 166aa, as shown in [77]. These thioredoxins perform different functions when released extracellularly, like getting involved in growth stimulation and chemotaxis, further acting as an antioxidant and regulator for transcriptional activity when present in the cytoplasm and nucleus, respectively [78].

Post-translational modification of Thioredoxin: Trx-1 is mainly localized in the cytosol but can be secreted out of the cells in two forms, a full-length Trx-1 and a truncated form called Trx-80 and the latter lacks redox activity but stimulates peripheral blood mononuclear cells (PBMC). Trx-80 contains the initial protein's first 80 or 84 N-terminal amino acids. It is a potent cytokine for monocytes and promotes a Th1 response through IL-12 production. TrxR cannot reduce Trx80 because Trx80 lacks the C-terminal portion of Trx, which is crucial for its interaction with Trx reductase. Hence, its role is crucial in reducing oxidative stress is confounding because of its potential to contribute to inflammation. Also, Trx1's (which can be reduced by TrxR and play major role in decreasing oxidative stress)enzymatic activity is decreased by glutathionylation under oxidative stress, but it later seems to regain it, indicating that Trx1 can de-gluthionylate itself by some autoactivation mechanism [70, 79], Table 2 and Fig. 4.

**Table 2:** Various modifications and interactions regulating thioredoxin activity

Post-translational modification	Amino acid involved	Function
Post-translational modifications that regulate thioredoxin activity		
1. Glutathionylation	Cys-73 in eukaryotic cells and cys-60 in plants	Significant reduction in Trx activity
2. Thiol- oxidation of Trx	Cys-32, 39 in redox regulatory domain	Binds to different proteins and regulates their protein functions
3. S-nitrosylation	Cys-69 and Cys-73 outside the regulatory region	Increased activity of thioredoxin and accounting for anti-apoptotic capacity of Trx
Thioredoxin interacting protein that regulates Trx functions		
1. TXNIP	Cys-247 of TXNIP interacts with thioredoxin	Inhibit thioredoxin
2. ASK-1	Trx binds to the N-terminal of ASK-1	Reduced thioredoxin binds ASK1 to inhibit downstream signaling
3. PKC	Binds to the catalytic domain of thioredoxin	Thioredoxin inhibits PKC-mediated histone phosphorylation

### Working mechanism of thioredoxin

**Fig. 6:**
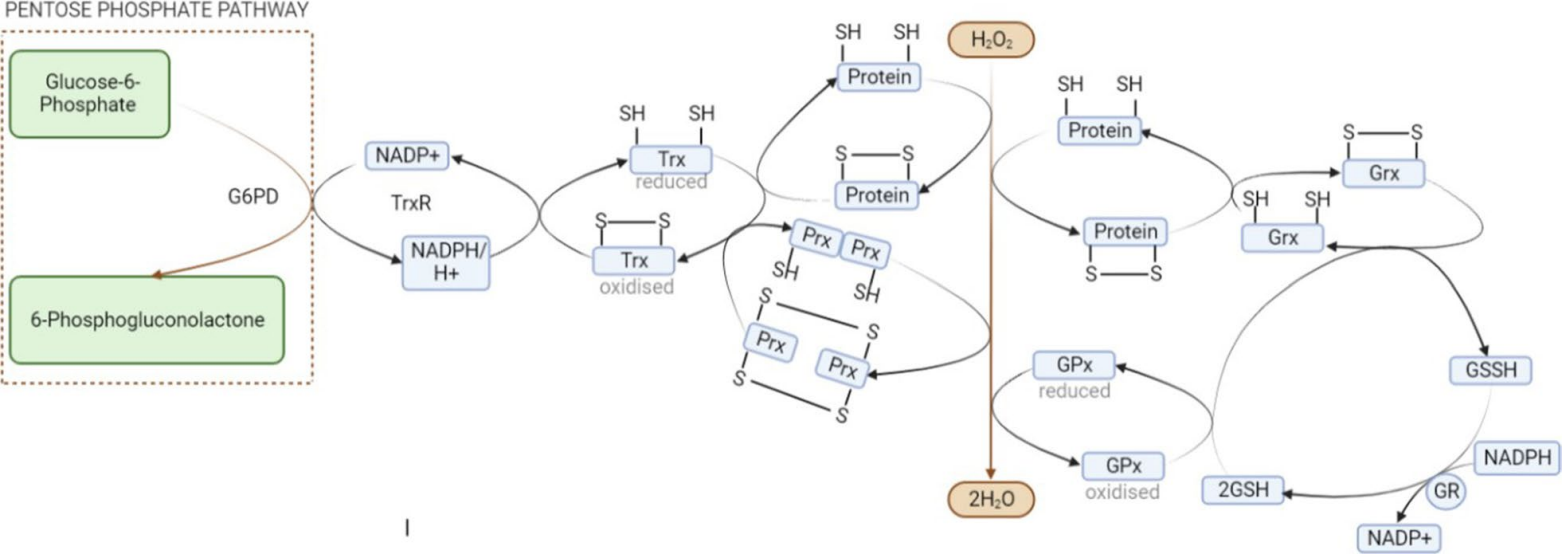
Crosstalk of thioredoxin with other antioxidants. Peroxidoxin can reduce the hydrogen peroxide after which it is reduced by Thioredoxin, while thioredoxin reductase (TrxR) reduces oxidized Trx in a NADPH-dependent manner. GPX combines the oxidation of GSH with the reduction of Hydrogen peroxide. Glutathione reductase (GR) reduces oxidized glutathione disulfide (GSSG) in the presence of NADPH

Electrons (including protons) are transmitted from NADPH to the flavo- and selenoprotein Thioredoxin reductase, which transfers them to the oxidoreductase Trx, which is utilized to reduce disulfides in target proteins substantially, as shown in Fig. 6 [80]. Trx possesses two cysteine residues actively engaged in oxidoreductase reactivity dithiol/disulfide exchange (in human, Thioredoxin-1 contain Cys-35 and Cys-32). The Cys-32 thiol group forms covalent disulfide linkages with the substrate protein (X-S2) through a hydrophobic nucleophilic process. Proton abstraction then allows reduced protein substrate to be released. TrxR and NADPH reversibly decrease the oxidized Trx-1, allowing new cycles to commence [81]. Other cysteines, including Cys73, Cys 62, and Cys69, have recently been discovered in Trx-1, and when Trx-1 becomes highly oxidized, these conventional cysteines form a second disulfide between Cys62 and 69. When Cys73 is further oxidized, it forms a homodimer with two molecules [82]. Lipopolysaccharides, hypoxia, H_2_O_2_, photochemical stress, and infections can activate Trx. Trx modulates oxidative stress and adaptive inflammatory reactions in several cell types, including type II pneumocytes, bronchial epithelial cells, and macrophages. Thioredoxin (Trx) is an anti-inflammatory and antioxidant cytokine that participates in various inflammatory conditions; many diseases, including pulmonary disease, are associated with OS. Trx-1 expression is elevated in respiratory illnesses such as acute (ALI), lung injury, cystic (CF) fibrosis, lung cancer, asthma, COPD, idiopathic (IPF) pulmonary fibrosis, and lung transplant rejection [83].

### Thioredoxin as a master regulator in realigning COPD-impaired pathways

The respiratory system and the lungs are consistently subjected to external stimuli such as toxins in the atmosphere and viral infection, including cigarette smoke and pollutants, resulting in the generation of ROS and oxidative tissue damage. These ROS, reactive nitrogen species (RNS), have been linked to the development of various lung illnesses in both animal and human models [84]. Thioredoxin, including TrxR (Thioredoxin reductase), has been displayed by alveolar macrophages and bronchial epithelium in a healthy lung [85]. The elevation of thioredoxin is viewed as an adaptive response to lung inflammation associated with oxidative stress [86], and direct linkages between OS and the pathophysiology of many lung disorders have been demonstrated [87]. Current smokers had significantly higher serum thioredoxin concentrations [88]. Several respiratory illnesses have been linked to increased thioredoxin levels in plasma/serum or bronchoalveolar (BALF) lavage fluid [89]. A study has found that individuals with acute exacerbations of chronic obstructive pulmonary disease (AECOPD) have lower serum levels of Trx1. According to the study, 4-hydroxy-2-nonenal (4HNE)-protein adducts dynamically increase as the partial pressure of oxygen (PaO2) decreases in the serum of AECOPD patients, while thioredoxin reductase (TrxR1) and thioredoxin (Trx1) steadily decline in comparison to those of healthy individuals. Another study found that Trx1 has anti-oxidative and anti-inflammatory capabilities, can reduce neutrophilic inflammation via anti-chemotactic actions, and can prevent emphysema from cigarette smoke. The research also suggests that Trx1 might help prevent COPD exacerbation [90]. A study further revealed that young and adult mice overexpressing Trx1 exhibited enhanced tolerance to oxidative stress and decreased oxidative damage to proteins and lipids. On the other hand, the transgenic mice only exhibited a 5.5% increase in longevity in the middle stages of life (25% survival), and no increase beyond that (10% survival), which was connected to decreased Trx1 overexpression. In a different study, only the first male cohort of the Tg(act-TRX1) mice significantly outlived the wild-type mice in the early part of life (75% survival), although the male mice in general showed a considerable increase in lifespan in the very early part of life (90% survival; both cohorts) [91]. As indicated in Table 3, numerous investigations have demonstrated that Thioredoxin can rectify the deteriorated pathways compromised in COPD.

**Table 3:** Summary of published studies on the outcomes of thioredoxin supplementation

Mechanism regulated by Trx	Model	Mode	Outcome	References
Oxidative stress	Mice	Intravenously administered	Through its redox-regulating function, TRX has specific cytoprotective effects on cerebral ischemia/reperfusion injury in mice	[119]
	Mice	Intraperitoneal administration	Through a variety of mechanisms, including scavenging reactive oxygen and nitrogen species and restoring endogenous Trx-1 levels, rhTrx-1 protects against APAP-induced liver damage	[120]
Inflammation	Rat	Intraperitoneal administration	Through an anti-chemotactic effect, rhTRX reduces LPS-induced bronchoalveolar neutrophil infiltration. In the xenotransplantation paradigm, administration of rhTRX had no effect on chemosensitivity or tumour growth	[121]
	Mice	Intraperitoneal administration	Trx-1 reduced GM-CSF secretion, which reduced neutrophilic inflammation	[104]
Protease- antiprotease	Mice	Intraperitoneal administration	The progression of emphysema was markedly delayed by TRX1 medication commencing 14 days after elastase administration	[102]
NF-kβ	HEK293 and HeLa cell lines		At the level of or downstream from TRAFs, TRX expression and antioxidant treatment inhibit inflammatory cytokine-induced NF-kB-mediated gene induction	[122]
	HMDMs		By acting on cell membrane surface receptors to restrict NF-B activation and translocation into the nucleus, extracellular Trx limits the formation of p50 and p65 and promotes I-B synthesis, ultimately limiting the NF-B pathway	[123]
PI3-Akt	Mice	Transgenic mice	Transfection of the Trx-1 gene or administration of recombinant human Trx-1 (rhTrx-1) decreased the cytotoxicity caused by indomethacin in rat gastric epithelial RGM-1 cells. Pre-treatment with rhTrx-1 reduces the formation of ROS and downregulates phosphorylated Akt caused by indomethacin	[118]

#### Thioredoxin realigning disrupted redox balance in COPD

There are different vital systems for maintaining redox homeostasis, out of which Trx is an essential regulator for maintaining a reduced cellular environment [92]. It has also been seen that when human Thioredoxin gets overexpressed in transgenic mice, it reduces the OS damage been seen that reduces the OS damage and extends the life span. Another life span factor, telomerase activity, was considerably less in wild mice than thioredoxin transgenic mice [93]. Even the transgenic mice that overexpress thioredoxin resist oxidative damage [94]. Due to its redox-active cysteine residues, the dithiol Trx is essential for maintaining a reduced cellular environment.TrxR transfers electrons from NADPH to the active region of Trx, which reduces substrates like protein disulfides. Through thiol-disulfide exchange processes, reduced Trx lowers proteins containing disulfide linkages by transferring electrons from their reactive cysteines [95].

Previously, TrxR was the only reductant; however, when TrxR was blocked by siRNA or the inhibitor aurothioglucose, no change in Trx1 redox state or cell viability was observed. Further investigation reveals that the GSH was acting as a TrxR in its absence, with the GSH and Glutaredoxins (GRX) systems acting as a backup for Trx and jointly directing the redox system [96]. Also, the Thioredoxin interacting protein (TXNIP), a down regulator of Thioredoxin function, is an important signalling molecule that reacts with Trx [97]. TXNIP’s Cys63 and Cys247 can create a mixed disulfide bond with the thiol group on Thioredoxin's active site, thus inhibiting Trx activity and causing oxidative stress [98]. Under normal circumstances, TXNIP is found in the nucleus with Poly (ADP-ribose) polymerase 1 (PARP1). TXNIP will migrate into the mitochondria or cytosol in response to oxidative stress, where it binds and oxidizes Trx1/Trx2 (redoxisome complex), lowering Trx1/Trx2 binding to ASK1 and initiating a signalling cascade controlled by apoptosis signal-regulating kinase 1 (ASK1) and hence ASK1 mediated apoptosis [99]. TXNIP translocation is also linked to increased ROS in mitochondria and the activation of the NLRP3 (NOD-, LRR- and pyrin domain-containing protein 3) inflammasome, which induces IL-1β expression, thus causing inflammatory reactions [100].

#### Thioredoxin rebalances protease-antiprotease equilibrium

According to studies, Trx and dihydro (DHLA) lipoic acid has been seen to reduce neutrophil elastase activity in humans. Elastase contains four disulfide bridges necessary for the enzyme's three-dimensional structure and activities. Even though the route whereby the Thioredoxin, including DHLA, limits the functioning of elastase was not elucidated in the investigation, such powerful reducing agents are expected to destroy disulfide connections in elastase. Because oxidized Trx and lipoic acid do not efficiently block elastase, the availability of reduced sulfhydryl molecules might seem to be a crucial aspect of this process [101]. The differential ability of Thioredoxin to hamper MMP and TIMP activity can help in reregulating the balance between TIMP and MMP, as shown in Fig. 2. As a result, the research uncovers a potential extrinsic function for the Trx/TrxR antioxidant defence system in the differential regulation of MMP and TIMP behaviour, as well as a potential method for altering the TIMP/MMP equilibrium [102].

#### Thioredoxin as an anti-inflammatory performer in COPD

Thioredoxin-1 contains antioxidant and anti-inflammatory capabilities, thus helping reduce neutrophil-induced inflammation via preventing chemotaxis and emphysema caused by CS [103]. Mice exposure to cigarette smoke, then afterwards medicated with polylysine- [poly (I: C)] showed increased OS, airway neutrophilic inflammation, and lung apoptosis in C57Bl/6 mice, which is smoke-sensitive, but not in New Zealand White inbred strain (NZW) mice (smoke-resistant), according to a study. In C57Bl/6 mice, Cigar smoke and poly (I: C) exposure increased the progression of emphysema. Thioredoxin-1 inhibited neutrophilic inflammation and the progression of emphysema. In the lungs exposed to CS, early neutrophilic inflammation was caused by poly (I: C) via releasing keratinocyte-derived chemokine (KC) and granulocyte/ macrophage colony-stimulating factor (GM-CSF). Persistent GM-CSF secretion caused late neutrophilic inflammation, which Trx-1 alleviated. Trx1 increased MAP kinase (MKP-1), phosphatase one pulmonary mRNA expression of and inhibiting MKP-1 reversed thioredoxin-1’s suppressive effects on persistent GM-CSF production and delayed neutrophilic inflammation [104]. Furthermore, Trx modulates neutrophil, monocyte, and lymphocyte migration. Trx, on the other hand, reduces the start and onset of acute respiratory (ARDS)distress syndrome and emphysema in humans [105]. Small molecule thioredoxin mimetics to treat an insulinoma cell line, *i.e.,* INS 832/13 cells, reduces cell death caused by the thioredoxin reductase inhibitor auranofin. MIN6 insulinoma cells have been shown to release thioredoxin 1 (Trx1) in low oxygen circumstances, and externally injected Thioredoxin-1 rescues MIN6 cells against hypoxia-induced death. According to this research, exogenous Thioredoxin enhances cell survival under environmental stressors through protein overexpression or peptide mimetics [106]. Thioredoxin suppresses the invasion and upregulation of inflammatory cells like neutrophils, which reduces the generation of inflammatory molecules, including the inflammatory response, as demonstrated in Fig. 2. CD62L is a neutrophil-shed molecule that helps neutrophils adhere to the endothelium of blood arteries and penetrate them. Trx inhibited CD62L adherence to endothelial cells in neutrophils and tried to prevent LPS-induced knockdown of CD62L exfoliation, while the ThioredoxinC32S/C35S mutant has no impact on neutrophil adherence with endothelial cells. It was observed that when the rat model induced with LPS received systemic thioredoxin dosage, it significantly reduced neutrophil recruitment throughout bronchial lung tissues; however, it did not specifically diminish the surge in endothelial LPS-induced (ICAM-1) intercellular adhesion molecule-1 cells. Finally, Thioredoxin can considerably minimize or heal tissue injury by reducing neutrophil recruitment and stimulation, as shown in Fig. 2 [107].

Thioredoxin suppresses eosinophil infiltration and activation by controlling the cellular signal route, the exogenous Th1/Th2 balance, and the molecule's interplay with Eosinophil-induced cytokine. Thioredoxin reduced ERK 1/2 and p38 mitogen-activated protein kinase activity by inhibiting eotaxin-induced activation of extrinsic signal-regulated kinase 1/2 and p38 mitogen-activated protein kinase activity. TRX inhibits airway remodelling in the lungs via inhibiting the synthesis of the chemokines MIP-1 and eotaxin and affecting T-helper2 cytokines like interleukin-13, which cause APCs to release eotaxin [108].

In response to increased Th2 cytokine responses, Trx increases the synthesis of Th1-like cytokines such as IL-1, Interleukin-1Receptor α, and Interleukin-18, inhibiting Th2-like cytokine expression. Lymphocytes taken from Trx-Transgenic mice can form T-helper2 cytokines like IL-5, IL-13, and IL-4 after they start leaving the increased Trx situation in vivo; Trx did not affect proliferation and differentiation of Th1/Th2, instead restricting inflammation by governing the formation and release of Th1/Th2 cytokines. Macrophage migrating (MIF) inhibitory factor expression was also reduced considerably in Trx-Tg lung tissue. Additionally, activated eosinophils produce TGF-β, which interacts with the epidermal growth (EGFR) factor receptor outside airway epithelial cells, prompting them to produce mucin connected to tracheal inflammation. Trx overexpression reduces EGFR synthesis and activation in cells. Trx controls eosinophil activity and migration, which limits allergic responses [109].

#### Thioredoxin in fixing NF-kB performance

**Fig. 7:**
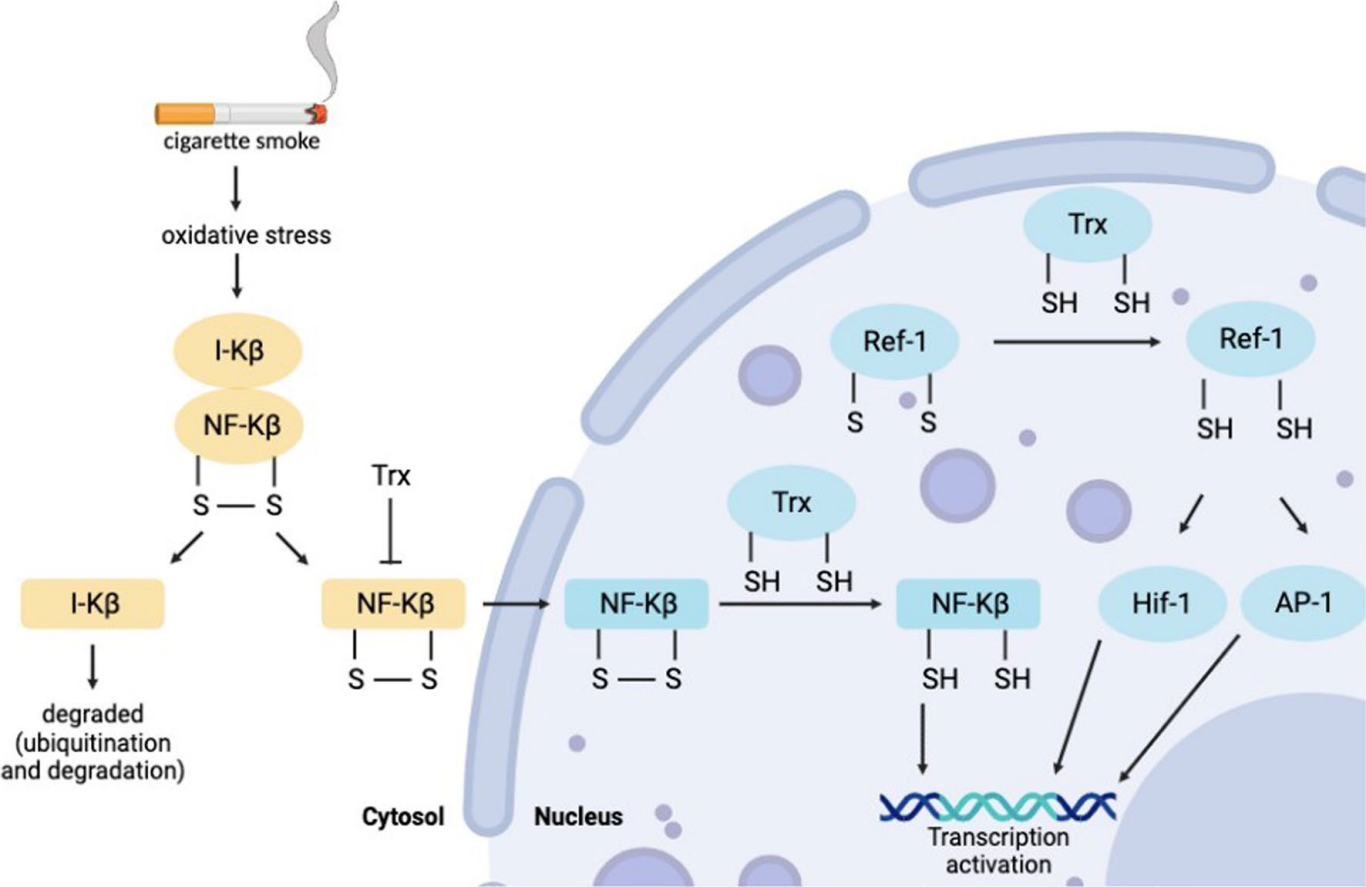
Thioredoxin regulating NF-KB pathway. In a normal scenario NF-KB is in an inactive state, but when exposed to oxidative stress, it activates and enters the nucleus to start the transcription of inflammatory genes. However, thioredoxin has been observed to suppress the ubiquitination of I-KB, which in turn inhibits the insertion of NF-KB into the nucleus

It is interesting to note that Thioredoxin has been demonstrated to prevent I-κβ degradation in the cytoplasm and can prevent NFKB translocation. At the same time, it also improves NF-κB activity by boosting its potential to adhere DNA in the nucleus, as shown in Fig. 7 [110]. TLR (Toll-like receptor) interacts with MyD88 that stimulates the IKK complex, which involves MAPK like p38, Erk), as well as c-Jun (JNK)N-terminal kinase, which then affects the interaction of the (transcription factor) TF like cAMP-response element binding protein (CREB), AP1, and NF-κB, leading to abnormal pre-inflammatory reaction in Txnrd1-deficient BMDCs. Furthermore, it was observed that robust TLR response in Txnrd1-deficient BMDCs after inducing with LPS or R837, as measured by phosphorylation and subsequent destruction of IkB-α and phosphorylation of Erk1/2, implies that the absence of the Trx1 system does not affect early downstream events after TLR stimulation. When Ikβ-α is proteolyzed, NF-κB p65 and p50 are released and translocated to the nucleus, promoting the transcription of pro-inflammatory cytokine genes. Then, the transportation of NF-κB inside the nucleus was examined, but no decay problems of Ikβ-α were identified in the lack of Txnrd1. However, there were no marked differences in NF-κB p65 nuclear translocation between Txnrd1-sufficient and Txnrd1-deficient BMDCs. The microscopy analysis shows that Txnrd1 loss has little effect on NF-kB p65 nuclear translocation, indicating a downstream problem. Txnrd1 loss, they reasoned, might impair NF-κB p65 binding to its DNA response element. And found reduced NF-κB p65 DNA adherence to the pro-inflammatory genes Il12b, Il1b, and Il6 promoters.

But by interacting with cellular membrane receptor protein to restrict NF-κB activity and its transport into the nucleus, extracellular thioredoxin limits the formation of p-50 and p-65 and increases iκ-b synthesis, thereby limiting the NF-κB pathway [111–113]. In addition, several investigations have shown that trx interacts directly with the GC receptor, enhancing cell responsiveness to glucocorticoids [114].

#### Thioredoxin in reregulating PI3K-AKT pathway

As previously mentioned, PTEN on chromosome 10 is found to be the significant suppressor of class I phosphoinositide-3-kinases (PI3Ks). Cigarette smoke, followed by the production of ROS, downregulates PTEN (by creating a disulfide bond between the catalytic Cys124 and Cys71), enhancing AKT phosphorylation and vice versa. Thioredoxin appears to play a significant role in mediating this step-in cell. However, it has been discovered that Trx-1 is typically oxidized to dimers in the presence of high ROS, and its oligomers have also been seen when treated for extended periods with organic peroxides and hydroperoxides.

The oxidized Trx-1 made the Trx system less effective and prevented it from reducing the oxidized PTEN. Trx-1 and PTEN interact by creating a disulfide bond between their active site cysteines, Cys32 in Trx-1 and Cys212 in PTEN, which inhibits PTEN’s lipid phosphatase activity [115], as depicted in Fig. 3. According to a recent study, CuHP (Cumene hydroperoxide)-mediated PTEN oxidation is irreversible in cells but can be reversed by the exogenous Trx system, giving researchers ideas [116]. Thioredoxin redox status was maintained in ischemia–reperfusion (I/R) by increased amounts of thioredoxin but not mitochondrial Trx-2. This enhanced PGC (Peroxisome proliferator-activated receptor-gamma coactivator)-1α expression via AKT/CREB activation, which upregulated mitochondrial expression of genes, thus protecting from ischemia–reperfusion injuries [117]. Trx also reduces indomethacin-induced ROS generation and phosphorylated Akt expression in rat gastric epithelial cells [118]].

## Future directions

The rise in COPD incidence and death underscores the need for additional study into the biological mechanisms of COPD progression and exacerbations and the development of highly effective therapies with fewer off-target or on-target side effects. As a result, new therapy strategies that are safer and more efficient should be created. As shown in the sections above, TRX lowers lung damage in COPD by improving the balance between proteases and antiproteases, regulating oxidative stress and inflammation through transcription factors, including PI3-APK and NF-kB, and other mechanisms. However, these pathways need to be explored more concerning thioredoxin before using it as treatment, as current treatments for COPD are limited and even have side effects like immunosuppression. In contrast, Trx has demonstrated in numerous studies that it can attenuate emphysematous alterations in COPD animal models by lowering CS-induced oxidative stress and inflammation, even in mice that corticosteroids cannot control. Therefore, employing TRX transgenic mice or injecting recombinant TRX can be used in a future study to determine whether TRX is effective during acute exacerbations. There are currently viable sources for the production of thioredoxin protein, and many protein expression systems, such as yeasts, lactobacilli, algae, and plant cells, have been proposed with inflammatory properties that are comparable to those identified for purified recombinant(rhTrx) human thioredoxin.

Further research on TRX dose modalities for COPD, including oral and inhalation delivery, is required. At the same time, thioredoxin has shown cancer risk in both animal studies and human clinical trials. Hence, a need to be researched. There are many controversies to this because of unanticipated adverse effects; thus, it is suggested to carefully choose the subgroup of patients that is most likely to benefit from this treatment and has fewer risk factors for lung cancer, which is possible if markers that can assess TRX levels in significant will be researched. Also, these indicators can help figure out what stage of lung deterioration the intervention is most likely to be able to stop or delay. As thioredoxin levels in the blood rise in COPD patients while decreasing intracellularly, we consider Thioredoxin therapy very helpful. Therefore, research into thioredoxin as a COPD treatment could pave the way for new opportunities in the future.

## Conclusion

Chronic bronchitis, emphysema, and remodelling are all symptoms of COPD, a lung disease. COPD is becoming more common worldwide, yet few effective medications are available, and none prevents the disease from progressing or addresses all of the symptoms. The heterogeneity of COPD’s onset and progression has impeded the discovery of new treatments. Given the numerous potential therapeutic pathways that can be pursued, careful examination of COPD patient phenotypes/endotypes will be critical for personalized treatment options, and detailed knowledge of disease pathogenesis in patient subsets will ensure that these emerging therapies are targeted appropriately. Trx is significant in the treatment of COPD because it successfully prevents the onset and progression of the disease through various mechanisms. As a result, small molecule manipulating Trx activity in the lungs appears to be a promising therapeutic option in pulmonary diseases and can be used as a treatment for COPD in the future.

## Data Availability

Data analyzed during this study are provided in full within the published article.
